# Recent Advances in the Utilization of Chiral Covalent Organic Frameworks for Asymmetric Photocatalysis

**DOI:** 10.3390/molecules29215006

**Published:** 2024-10-23

**Authors:** Peng Liu, Weijun Dai, Xianfu Shen, Xiang Shen, Yuxiang Zhao, Jian-Jun Liu

**Affiliations:** 1 College of Chemistry and Environmental Science, Qujing Normal University, Qujing 655011, China; liupengxjlp@163.com (P.L.); xianfu_shen@163.com (X.S.); sx008100@163.com (X.S.); 2 School of Ethnic Medicine, Yunnan Minzu University, Kunming 650504, China; dwj1015@163.com

**Keywords:** covalent organic frameworks, asymmetric photocatalysis, chirality, synthetic method

## Abstract

The use of light energy to drive asymmetric organic transformations to produce high-value-added organic compounds is attracting increasing interest as a sustainable strategy for solving environmental problems and addressing the energy crisis. Chiral covalent organic frameworks (COFs), as porous crystalline chiral materials, have become an important platform on which to explore new chiral photocatalytic materials due to their precise tunability, chiral structure, and function. This review highlights recent research progress on chiral COFs and their crystalline composites, evaluating their application as catalysts in asymmetric photocatalytic organic transformations in terms of their structure. Finally, the limitations and challenges of chiral COFs in asymmetric photocatalysis are discussed, with future opportunities for research being identified.

## 1. Introduction

Photocatalysis is widely accepted as a convenient method in the field of asymmetric catalysis and has shown promise for bulk production [1,2,3,4,5,6]. Since MacMillan’s group reported in 2008 that photoredox catalysis combined with organocatalysis promotes asymmetric α-alkylation of aldehydes through a process catalyzed by ruthenium complexes [7], efforts have been made to develop more environmentally benign asymmetric photocatalysts and to extend their application to more meaningful reactions [8,9,10,11,12,13,14,15]. In recent years, hybrid photocatalysts consisting of inorganic semiconductor photocatalysts such as PbBiO_2_Br and [W_10_O_32_]^4−^ and chiral organic catalysts have shown excellent photocatalytic performance in stereoselective carbon–carbon bond formation [16,17]. These materials combine the advantages of the high stereoselectivity of organic catalysis with the stability and easy separation of heterogeneous catalysts [18,19]. However, related reports are still very limited, and designing new photocatalyst systems for asymmetric photochemical reactions is challenging.

In the past few years, an increasing number of studies based on reticular framework materials and their composites have been published in the field of photocatalysis, and their extraordinary features have attracted great attention [20,21,22]. Metal–organic frameworks (MOFs) are a promising reticular framework material for photocatalysis because they are composed of metal ions or clusters and organic ligands, which can act as active sites separately or cooperatively, affecting the light-harvesting, charge generation and separation, and surface reactions for remarkable photocatalytic performances [23,24,25]. At present, a large number of reviews focused on the application of MOFs in the field of photocatalysis have been reported [26,27,28]. Unlike MOFs constructed by coordination bonds, covalent organic frameworks (COFs) are a novel class of organic reticular framework materials, which are composed of organic building units linked only through covalent bonds, and they exhibit well-defined structures and permanent porosity. Crystalline COF materials hold potential for applications in energy storage, optoelectronics, catalysis, gas storage and separation, and drug delivery [29,30,31,32,33,34,35,36]. It is well known that the chirality of molecules can be amplified by a reticular structure [37,38]. In addition, the enantioselectivity of asymmetric transformations can be improved by fixing chirality into constrained framework spaces. Therefore, introducing chiral functional groups and photoactive units into COFs through a rational selection of organic monomers can pave the way for chiral functionalities to have viable applications, such as in asymmetric photocatalysis.

In 2014, Jiang and co-workers reported the first chiral COF and its application in asymmetric catalysis [39]. Since then, numerous chiral COFs have been created, and their diverse applications in asymmetric catalysis, chiral separation, and enantioselective sensing have been investigated [40,41,42,43,44]. Recently, Cui et al. published two seminal reviews on chiral COFs [45,46], concentrating on their synthesis, structural features, and applications. Although a large number of examples of chiral COFs and some reviews of their applications have been reported, the construction of chiral COFs with chiral photocatalysts is still a challenge due to the need to consider both chiral catalysis and photocatalysis and their synergistic relationship. To date, a comprehensive review outlining the synthesis and structures of chiral COFs and their applications in asymmetric photocatalytic organic transformations has not been published.

This review mainly focuses on summarizing the recent research progress on chiral COFs and their crystalline composites for asymmetric photocatalytic organic transformations evaluated in terms of their structure. The photochemical properties and asymmetric photocatalytic performance of these materials are illustrated to provide a useful guide for the preparation and application of chiral COFs in the future.

## 2. Synthesis and Structure of Chiral COFs

Since Yaghi’s group first reported COF powders in 2005, many research groups have employed a variety of synthetic methods to prepare COFs [47]. To date, COFs are mainly synthesized by solvothermal [48,49,50], microwave-assisted [51,52], ionothermal [53,54], flux synthesis [55], and room temperature methods [56,57,58]. Despite the success of these approaches, the first chiral COF was not synthesized until 2014 [39]. A major challenge in chiral COFs is balancing the asymmetry of chiral monomers and the crystallinity of materials because of the low-symmetry nature of chiral monomers, which contradicts with the observations that most COFs are crystallized in high-symmetry space groups, with multiple crystallographic symmetry elements residing on each monomer. Moreover, in the preparation of chiral COFs with specific functions, the solvent and temperature are crucial factors in obtaining crystalline products.

**Figure 1:**
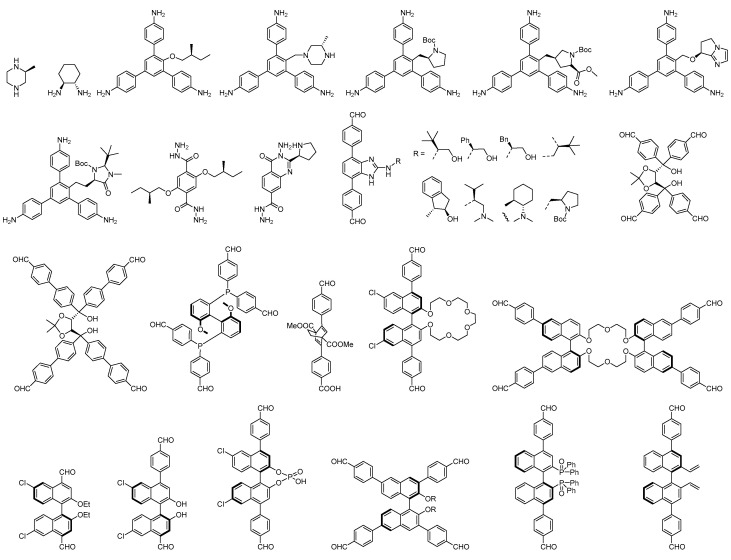
The chemical structures of chiral monomers used in the construction of chiral COFs.

To date, all chiral COFs have been synthesized by the solvothermal method. However, the solvothermal synthesis of chiral COFs can also be categorized into the following three different synthesis approaches [59,60,61,62,63,64]. First, in de novo synthesis, homochiral COFs are prepared using either enantiopure monomers or achiral monomers with chiral crosslinking units. Second, in a post-synthesis approach, achiral COFs are modified at the level of organic monomers or by inserting chiral substances into the pores. Third, in a chiral induction approach, achiral monomers are used as precursors in the chiral catalytic or chirality-induced synthesis of homochiral COFs. In this section, we briefly introduce the various strategies and synthetic conditions for producing chiral COFs and describe the relationship between their structure and properties. Figure 1 shows the structures of chiral monomers used to construct chiral COFs via the de novo synthesis method.

### 2.1. De Novo Synthesis

Constructing predesigned chiral COFs through the polymerization of rationally selected enantiopure monomers, guided by framework chemistry design rules, is a promising yet challenging approach. The inherent chirality of the building blocks is preserved in the overall framework, maintaining the absolute configuration of the resulting homochiral structures [65]. The inherent chirality of the building blocks is conserved in the absolute configuration of the resulting homochiral framework, and chiral COFs synthesized by de novo synthesis exhibit a well-defined structure and uniformly distributed chiral sites. Chiral COFs can be categorized into two structural types: (1) a structure in which the framework itself is chiral and (2) a structure in which the framework is achiral but the substituents on the framework are chiral. However, both of them crystallize in chiral space groups.

In 2016, Wang and colleagues reported the first de novo synthesis process [66], preparing two chiral COFs, LZU-72 and LZU-76, which crystallized in the *P*3 space group and exhibited a 2D layered hexagonal network with high crystallinity. The frameworks of these COFs are achiral but the substituents on the framework are chiral. The chiral monomer was synthesized from rigid 4,4′-(1*H*-benzo[*d*]imidazole-4,7-diyl)dianiline with chiral pyrrolidine substituents. The resulting chiral COFs are structurally robust and highly active as heterogeneous catalysts in asymmetric aldol reactions. Subsequently, Wang’s group explored a general method for introducing different chiral functional groups into an achiral COF [67]. They used the achiral molecule DBCBI (4,7-dibromo-2-chloro-1H-benzo[*d*]imidazole) as a platform on which to immobilize eight chiral functional groups via nucleophilic substitutions and Suzuki coupling to obtain eight chiral monomers. These chiral monomers were condensed with 1,3,5-tri(4-aminophenyl) benzene (TAPB) to produce eight 2D chiral COFs, which crystallize in the *P*3 space group with a hexagonal network. The high-throughput synthesis of chiral COFs from a platform molecule not only provided eight catalysts for the asymmetric reaction of β-ketone esters, but also offered a general method for structure–activity studies of COFs.

In 2016, Yan et al. reported a bottom-up strategy for the synthesis of chiral COFs [68]. First, they prepared a C_3_-symmetrical 1,3,5-triformylphloroglucinol (Tp) to bind linear chiral carboxylic acids. Then, this chiral building block was directly condensed with benzidine (BD), 1,4-phenylenediamine (Pa-1), and 2,5-dimethylp-phenylenediamine (Pa-2) affording the 2D hexagonal chiral COFs CTpBD, CTpPa-1, and CTpPa-2, respectively. On this basis, they used an in situ growth approach to prepare chiral COF-bound capillary columns for chiral separation.

Another report by Zhang’s group described a hydrazone-linked chiral COF based on 1,3,5-benzene-tricarboxaldehyde with chiral (*S*)-2-methylbutoxy groups [69] that showed good chemical stability and high performance for separating enantiomers. At the same time, Liu, Cui, and co-workers explored a multivariate method to prepare several multi-component 2D homochiral COFs based on 1,3,5-tris(4-aminophenyl)benzene with chiral l-imidazolidine and l-imidazolidine side chain modifications on the central aromatic ring [70]. These chiral COFs were successfully obtained by the multi-component crystallization of two dialdehydes and triamines with or without chiral organic catalysts.

**Figure 2:**
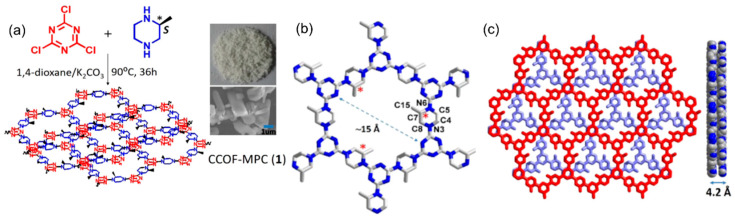
(**a**) A schematic diagram of the preparation of CCOF-MPC; (**b**) a single-layer hexagonal building unit of CCOF-MPC; (**c**) the stacking patterns of CCOF-MPC. Adapted from ref. [71] with permission, copyright 2017, the American Chemical Society.

Using a similar strategy, Dong’s group synthesized a chiral COF in 2017 by directly condensing the chiral monomer of *S*-(+)-2-methylpiperazine (S-MP) and cyanuric chloride [71]. The cyanuric chloride is connected by S-MP and extends on the *ab* plane to form a 2D layer network (CCOF-MPC) with hexagonal cavities (Figure 2). The chirality of this chiral COF is derived from the chiral S-MP affixed to the inner wall of the COF channel. The crystalline CCOF-MPC is then subjected to Pd(NO_3_)_2_ solution impregnation and metal reduction steps to obtain the final metal nanoparticle-loaded chiral composite (Pd@CCOF-MPC). Pd@CCOF-MPC can effectively promote the Henry reaction and reductive Heck reaction in a high yield with excellent stereoselectivity.

Chiral COFs are prepared using achiral skeleton molecules. By nucleophilic substitution or condensation, the chiral centers are modified or attached to the surface or inner wall of the COFs, but the COF framework remains achiral. To further release the chiral expression potential of the materials, in 2018, Cui’s group reported two chiral COFs with chiral scaffolds using enantiopure monomers TADDOL (tetraaryl-1,3-dioxolane-4,5-dimethanol) and tetra(4-anilyl)methane (TAM) [72]. The TADDOL-based *tetra*-aldehydes react with a tetrahedral TAM to form 3D imine-based chiral COFs with permanent porosity and high crystallinity. Powder X-ray diffraction, computer simulation, and an aperture distribution analysis indicated that both chiral COFs crystallized in the *P*2 space group and can be used as chiral stationary phases for high-performance liquid chromatographic enantioseparation.

BINOL is a crucial chiral compound in organic chemistry and material science. However, it was not until Cui’s group synthesized two BINOL-based chiral COFs that the application of chiral COFs was realized. They achieved this by designing enantiopure BINOL-based dialdehydes with 1,3,5-tris(3,5-diisopropyl-4-aminophenyl)benzene (CCOF 7) and tetrakis(4-aminophenyl)ethane (CCOF 8) [62]. In this work, CCOF 7 crystallized in the *C*2 space group with 2D layered tetragonal networks. CCOF 8 crystallized in the *R*3 space group, exhibiting a staggered stacking mode of 2D hexagonal layered frameworks. Notably, due to weak interlayer interactions, CCOF 7 can be easily exfoliated into two-dimensional sheets and exhibits superior capabilities for the detection of chiral odor vapors in both the solution and membrane. In 2021, the same group reported two 3D chiral COFs that have 9-fold or 11-fold interpenetrated diamondoid frameworks by condensing the tetrahedral tetramine and enantiopure BINOL [73]. The chiral COFs demonstrated exceptional asymmetric catalytic activities for the cyclocondensation of aldehydes and anthranilamides into 2,3-dihydroquinazolinones. This is attributed to chiral BINOL units that are uniformly distributed within the tubular channels, which facilitate preferential secondary interactions between the framework and substrate, thereby inducing enantioselectivity.

In addition, Dong et al. synthesized a chiral COF using a gold *N*-heterocyclic carbene (NHC-Au) building block and chiral secondary amine through a modified de novo synthesis approach [74]. The chiral COF (*S*)-NHC-Au-SA-COF is a heterogeneous catalyst for the asymmetric aryl methanol oxidation-aldol relay reaction. Moreover, this chiral COF-based setup was developed using a facile templating freeze-drying method based on an eco-friendly chitosan material, promoting the highly asymmetric aerobic alcohol oxidation-aldol relay reaction on a gram scale.

**Figure 3:**
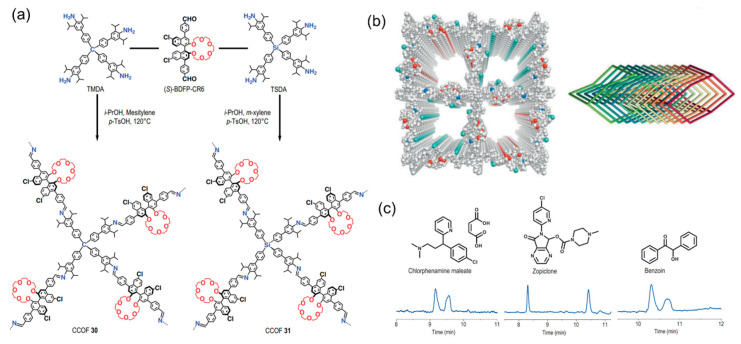
(**a**) Construction of 3D chiral COFs; (**b**) 3D structure and 11-fold interpenetration of diamond framework; (**c**) chiral crown ether-decorated COFs are used for separation of racemates. Adapted from ref. [75] with permission, copyright 2024, Oxford University Press.

Recently, Cui’s group designed two 3D chiral COFs via the polycondensation of a chiral 1,1′-binaphthyl-20-crown-6-derived dialdehyde and tetraamines with diisopropyl substituents (Figure 3a) [75]. Structural characterization showed that both COFs had 11-fold interpenetrating diamond frameworks characterized by tubular open channels decorated with chiral crown ethers as enantioselective recognition and binding sites (Figure 3b). These chiral COFs are particularly useful for the chiral separation of epoxides, ketones, and drugs when applied as coatings for chiral columns (Figure 3c).

**Figure 4:**
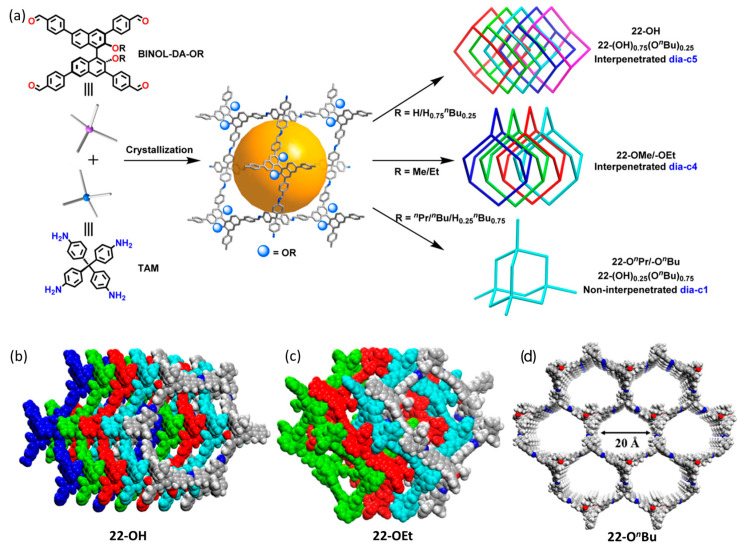
(**a**) Synthesis diagram of 3D chiral COFs with different alkyl substituents; (**b**) five-fold interpenetrated dia network of 22-OH; (**c**) four-fold interpenetrated dia network of 22-OEt; (**d**) noninterpenetrated dia network of 22-O*^n^*Bu. Adapted from ref. [77] with permission, copyright 2024, American Chemical Society.

In the past year, to further explore the properties of chiral COF materials, Cui and co-workers synthesized a series of chiral COFs with different topological structure types by a de novo synthesis method [38,76,77,78,79,80]. For example, they reported a mixed-linker strategy to design multicomponent 2D chiral COFs by the condensation of a mixture of triamines (with or without the MacMillan imidazolidinone catalyst or aryl substituent (ethyl and isopropyl)) and a dialdehyde derivative of thieno-[3,2-*b*]thiophene [76]. Structural characterization showed that these three-component homochiral 2D COFs feature either AA or ABC stacking. The stacking modes that can be synthetically controlled through steric tuning using different aryl substituents affect their chemical stability and electrochemical performance. With the MacMillan catalyst periodically appended on their channels, all of these COFs can be highly enantioselective and recyclable electrocatalysts for the asymmetric *α*-arylation of aldehydes, affording alkylated anilines with up to 97% enantiomeric excess by an anodic oxidation/organocatalytic protocol. Soon afterwards, they created a range of two- and three-component 3D chiral COFs by utilizing enantiopure BINOL-derived tetraaldehydes with varying alkyl substituents [77]. By imine condensations of BINOL-derived tetraaldehydes bearing different alkyl substituents with the monomer *tetra*(*p*-aminophenyl)-methane, a series of two-component and three-component chiral COFs with different interpenetrated dia networks were obtained (Figure 4a). Structural characterization showed that alkyl groups are appended on the walls of the channels, and their types/contents that can be synthetically tuned control the interpenetration degree of COFs by minimizing repulsive interactions between the alkyl groups (Figure 4b–d). This work provides a potential way to use steric hindrance to regulate and control the interpenetration, stability, porosity, and functionalities of chiral COFs.

**Figure 5:**
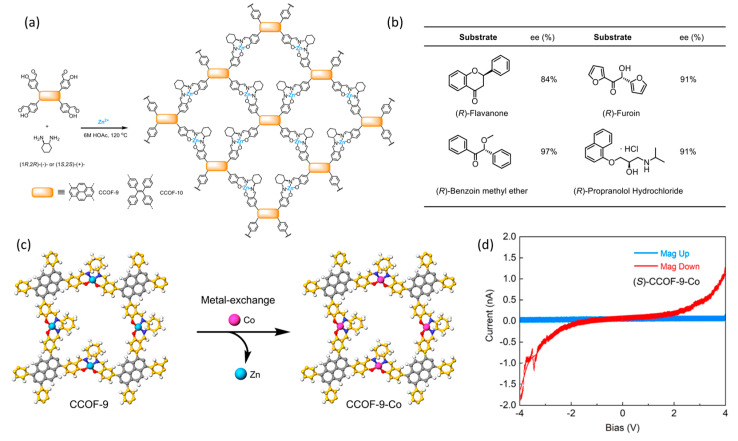
(**a**) Synthesis diagram of Zn(salen)-based chiral COFs; (**b**) enantiosorption of CCOF-9 toward racemic drugs; (**c**) synthesis diagram of CCOF-9-Co; (**d**) *I*–*V* curves of (*S*)-CCOF-9-Co obtained from mc-AFM. Adapted from ref. [79] with permission, copyright 2024, American Chemical Society.

The chirality-induced spin selectivity (CISS) effect describes a phenomenon wherein the charge transport through certain chiral structures or molecules exhibits a distinct chirality-dependent electronic spin polarization. The use of chiral COFs to achieve CISS remains a largely untapped area of research. More recently, Cui’s group synthesized four 2D Zn(salen)-based chiral COFs, namely CCOFs 9–12, by the imine condensation of chiral 1,2-diaminocyclohexane and *tri*- or *tetra*-(salicylaldehyde) derivatives (Figure 5a) [79]. CCOF-9 has a unique C2-symmetric “armchair” tetra-substituent pyrene conformation and exhibits the most pronounced chirality among these chiral COF materials, which means it can act as a solid state host capable of achieving the enantioselective adsorption of racemic drugs with up to 97% ee (Figure 5b). After paramagnetic Co(II) replaces diamagnetic Zn(II) ions, the obtained CCOF-9-Co not only maintains its high crystallinity and porosity and excellent chirality, but also exhibits enhanced conductivity (Figure 5c). Magnetic conductive atomic force microscopy shows that CMOF-9-Co has an impressive spin polarization ratio of 88–94% (Figure 5d). This work shows the great potential of chiral COFs for controlling spin selectivity and will motivate the development of novel crystalline polymers with the CISS effect. Using a similar strategy, they reported a one-step, template-free approach for synthesizing higher-order superhelical nanofibrous chiral COFs with preferred helicity by controlling alkyl chain lengths in organic linkers [80]. This approach yields chiral 3D COFs (13-OR, R = H, Me, Et, *^n^*Pr, *^n^*Bu) with a 10-fold interpenetrated diamondoid structure from enantiopure 1,1′-bi-2-naphthol (BINOL)-based tetraaldehydes and tetraamine. The superhelical nature of these chiral COFs is evident in their chiral recognition and spin-filter properties, displaying significantly enhanced enantiodiscrimination in carbohydrate binding and a remarkable CISS effect with a 48–51% spin polarization ratio. This work provides a robust and effective method for manipulating COF superhelical nanostructures, offering new insights into CISS in chiral materials.

According to the above research progress, the chiral COFs synthesized via the de novo synthesis method exhibit uniformly distributed chiral sites throughout the framework. Chiral scaffold COFs generally exhibit higher porosity and larger pore channels, whereas in chiral COFs with non-chiral scaffolds, a material porosity is typically reduced to a certain extent due to the chiral groups on the side chains of the framework. Therefore, the de novo synthesis of chiral COFs with chiral scaffolds has garnered significant attention, accelerating the rapid development of chiral COFs.

### 2.2. Post-Synthesis

The post-synthesis strategy is more commonly employed than de novo synthesis in the field of chiral porous solid materials. Post-synthetic modification can easily introduce chiral groups, accurately locate the chiral functions at anchor points of the achiral framework, and maintain the overall structure of the achiral framework [81,82]. Therefore, post-synthetic modification is an efficient and practical method for regulating the structure and function of COFs. This approach is ideal for achiral COFs with suitable functional groups, enabling the creation of functionalized chiral COFs by attaching the chiral component to the achiral COF framework through chemical bonds or non-covalent interactions.

**Figure 6:**
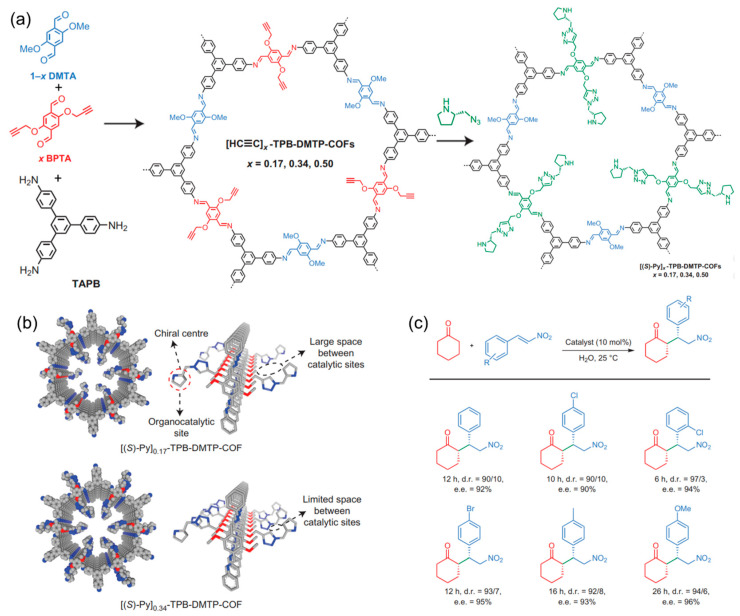
(**a**) Construction of chiral COFs through post-synthetic modification. (**b**) Channel wall structure of chiral COFs. (**c**) Substrate scope of Michael addition reactions catalyzed with chiral COFs. Adapted from ref. [83] with permission, copyright 2015, Nature Publishing Group.

The first chiral COF synthesized by post-synthetic modification, reported by Jiang’s group, was based on imine-linked porphyrins [39]. In 2014, they used the acetylene-azide click reaction to anchor optically pure pyrrolidine to the achiral COF framework, allowing the rational construction of the chiral COFs [Pyr]_x_-H_2_P-COFs (x = 0, 25, 75, and 100). The experimental results indicate that the modified COFs exhibit reduced BET surface areas and pore sizes compared to the parent COF, while the crystallinity remains unchanged. By regulating pyrrolidine loading on the pore walls, the modified COFs demonstrate superior catalytic activity for asymmetric Michael additions. Subsequently, the same group reported three chiral COFs using the same click reaction [83]. First, they synthesized an achiral 2D COF that was stable and resistant to strong bases and acids by the condensation of methoxy-containing terephthalaldehyde with 1,3,5-*tri*(4-aminophenyl)benzene (Figure 6a). The (*S*)-pyrrolidine was then anchored to the COF channel wall through the azide-ethyl click reaction, converting the achiral COF into a catalytically active chiral COF while maintaining the porosity and crystallinity (Figure 6b). As organocatalysts, the obtained chiral COFs show excellent activity toward Michael additions under ambient conditions, with over 90% enantioselectivity (Figure 6c).

In addition to the covalent binding of small organic molecules as chiral attachments, increasing numbers of examples have been obtained by grafting optically pure natural biomolecules such as sugars, enzymes, and peptides onto COF channel walls through immobilization and stabilization [44,84,85,86]. For example, Chen and Ma et al. proposed a general strategy for enriching chirality by covalently anchoring a range of biomolecules (lysine, tripeptides, and lysozyme) into achiral polyimide COFs [85]. The resulting biomolecule⊂COFs retain the framework structure of the parent COF but exhibit reduced pore sizes and surface areas. Interestingly, the biomolecule⊂COFs showed high chiral separation efficiency when used as chiral stationary phases due to the specific interactions and strong chirality inherited from the immobilized biomolecules. In the same year, Ma’s group incorporated optically pure lipase into achiral 2D COFs, creating chiral enzyme⊂COFs in phosphate buffer solution [42]. Compared to amorphous analogues, the porous channels of enzyme⊂COFs not only make the enzyme more accessible to the reagents, but also act as strong shields to protect the enzymes from inactivation, demonstrating superior activity and tolerance to harsh environments.

**Figure 7:**
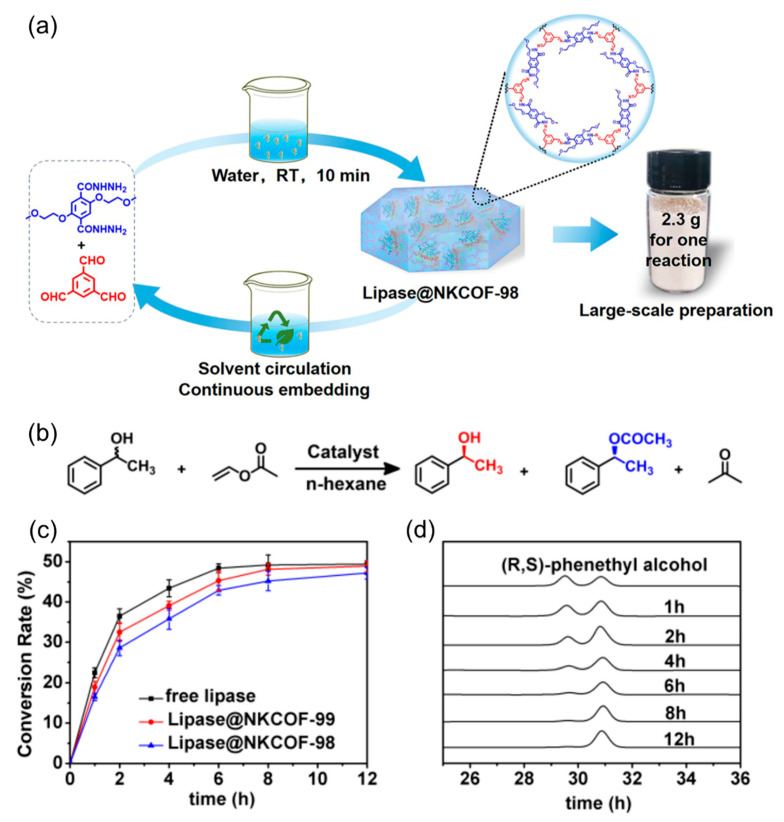
(**a**) The synthesis process of lipase@NKCOF-98 and a photograph of a lipase@NKCOF-98 sample from a scaled-up synthesis process. (**b**) The chiral catalytic resolution of 1-phenylethanol. (**c**) The conversion rate curves of different catalysts over 12 h. (**d**) The catalytic reaction results of NKCOF-99 at different reaction times (HPLC). Adapted from ref. [88] with permission, copyright 2022, Wiley-VCH.

Chen et al. have also made significant advancements in constructing chiral COFs by encapsulating bioactive molecules into achiral COF channel walls through post-synthetic modifications [87]. For example, in 2022, they introduced a scalable and green method for creating high-performance biocatalysts by assembling enzymes (lipase) with COFs under ambient conditions (Figure 7a) [88]. The obtained lipase@COFs demonstrated remarkable reusability and stability, serving as more effective catalysts for key industrial reactions than free enzymes or those immobilized by traditional methods (Figure 7b–d).

Cyclodextrins are a class of important biomolecules with intrinsic chiral cavities. In 2019, Cui et al. constructed chiral COFs by anchoring chiral β-cyclodextrin (β-CD) into channels of a 2D COF through a thiol-ene click reaction [89]. The modified COFs exhibited reduced pore sizes and surface areas compared to the parent COF while retaining the same crystal structure. Nevertheless, modified chiral COFs with low β-CD loading showed high enantiomeric discrimination of amino acids. Recently, Yi et al. constructed a carboxyl-functionalized achiral COF (TpBD-3COOH) with hexagonal channels and then integrated a chiral molecule, heptakis(6-amino-6-deoxy)-β-CD (Am7CD), to obtain a chiral COF (TpBD-Am7CD) [90]. Compared with TpBD-3COOH, the chiral selectivity of TpBD-Am7CD is significantly improved in adsorption experiments. At the same time, TpBD-Am7CD exhibited chiral selectivity to adsorb amino acid enantiomers similar to the previously reported β-CD COF, indicating that the chiral Am7CD functionality provides a chiral microenvironment.

The research described above indicates post-synthetic modification to be an effective and simple method for synthesizing chiral COFs. However, several shortcomings of the approach remain to be resolved. First, a uniform distribution of the chiral reagents introduced within the COF cannot be ensured. Second, the introduction of chiral functional groups inevitably leads to a decrease in the porosity of the COF. Third, the chiral appendants must be smaller than the COF channel diameter, limiting the scope of the approach.

### 2.3. Chiral Induction Synthesis

Chirality-induced synthesis is a highly appealing method employing chiral small-molecule catalysts to convert achiral reagents into optically active polymers, supramolecular systems, and other small molecules. Chiral induction has produced homochiral polymers with diverse application potential and has been applied in the construction of chiral COFs from achiral monomers.

**Figure 8:**
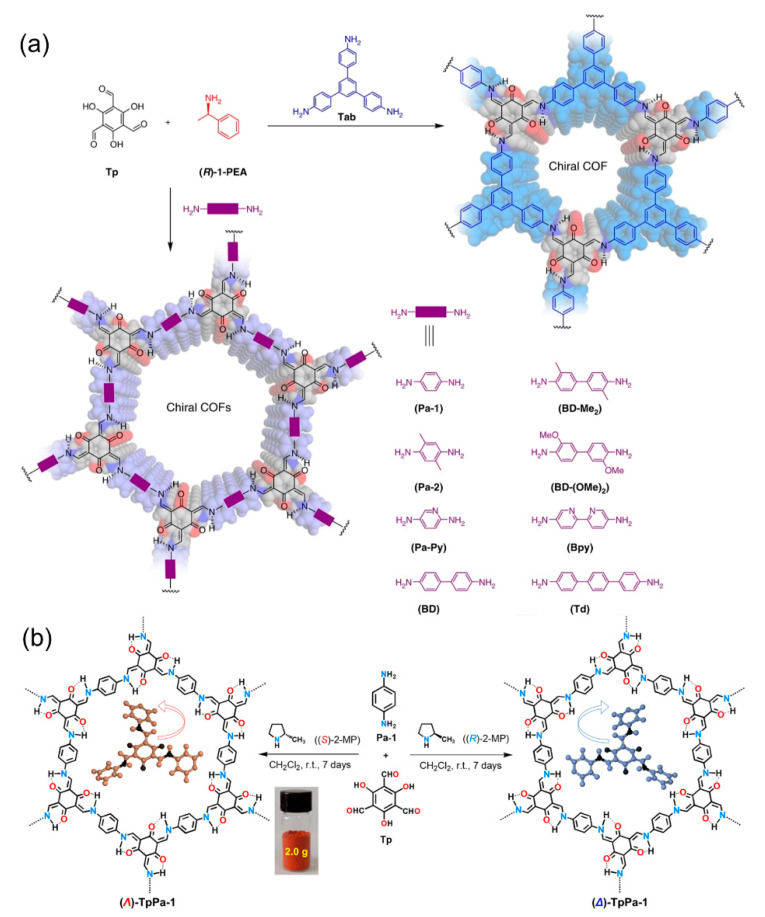
(**a**) Synthesis of chiral COFs by chiral 1-phenylethylamine asymmetric condensation. Adapted from ref. [91] with permission, copyright 2018, Nature Publishing Group. (**b**) Synthesis of (*Λ*)- and (*∆*)-TpPa-1 by chiral 2-methylpyrrolidine asymmetric polymerization. Adapted from ref. [92] with permission, copyright 2022, Wiley-VCH.

In 2018, inspired by metal–organic framework chiral induction studies, Cui’s group first used chiral induction synthesis to construct nine 2D chiral COFs [91]. These chiral COFs were solvothermally condensed from achiral *C*_3_-symmetric 1,3,5-triformylphloroglucinol (Tp) and triamine or diamine linkers in the presence of catalytic amounts of (*S*)- or (*R*)-1-phenylethylamine (Figure 8a). The chirality of these COFs is derived from the chiral catalyst-induced immobilization of tris(*N*-salicylideneamine) cores by intramolecular hydrogen bonding during crystallization. These chiral COFs showed high enantioselectivity toward chiral carbohydrates. Moreover, the post-synthetic modification of enaminone groups with Cu^2+^ enables metalated chiral COFs to serve as highly stereoselective heterogeneous catalysts for the asymmetric Henry reaction. Subsequently, Dong’s group prepared a chiral COF with high crystallinity via organocatalytic asymmetric Schiff base condensation under ambient conditions [92]. In this case, they employed chiral 2-methylpyrrolidine as a catalyst for the asymmetric condensation of tricarbonyl phloroglucinol with various hydrazides or diamines to prepare a series of tris(*N*-salicylideneamine)-derived *β*-ketoenamine chiral COFs (Figure 8b). Furthermore, the obtained chiral COFs can be metalated using a solid-state coordination method, providing heterogeneous catalysts for asymmetric A^3^-coupling reactions. Similarly, using chiral amines as chiral inducers, Gu et al. synthesized ultra-thin 2D chiral COF nanosheets from achiral monomers which exhibit tunable circularly polarized luminescence [93].

In 2020, Dong et al. prepared, for the first time, two homochiral COFs through the polymerization of achiral triamine dialdehydes and terminal aryl alkynes with the assistance of catalytic inducer CuOTf·toluene/(*S*,*S*)-pydox (pydox = 2,6-*bis*(4-phenyl-2-oxazolinyl)pyridine) [94]. The resulting propargylamine-linked chiral COFs can be highly reusable chiral catalysts of asymmetric Michael addition reactions with moderate activity and enantioselectivity.

Although achiral monomers can be assembled into chiral COFs by introducing chiral inducers or chiral catalysts, the chirality-induced synthesis of COFs is still in its infancy. To date, only a limited scope of chiral small molecules or chiral catalysts has been investigated. In addition, the development of alternative chiral inducers like circularly polarized light or chiral solvents is essential for advancing new chiral COFs, and the mechanism of chiral induction should be further studied.

## 3. Application of Chiral COFs in Asymmetric Photocatalysis

Asymmetric catalysis is one of the most efficient methods to obtain homochiral compounds. Photocatalytic organic synthesis utilizing sunlight is a green organic synthesis protocol offering the advantage of low energy consumption. The combination of these methods in asymmetric photocatalysis has attracted much attention in recent years, not least due to its application in drug synthesis [95]. Chiral COFs, as emerging porous materials, show significant potential as robust heterogeneous catalysts for asymmetric photocatalysis as they are readily functionalized and structurally regulated at the molecular level.

**Figure 9:**
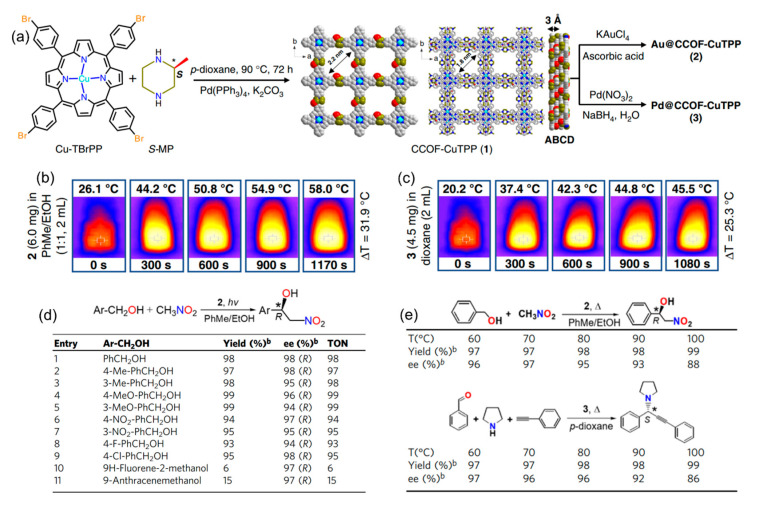
(**a**) Synthesis of Pd@CCOF-CuTPP and Au@CCOF-CuTPP. (**b**) Photothermal effect of Au@CCOF-CuTPP. (**c**) Photothermal effect of Pd@CCOF-CuTPP. (**d**) Scope of Au@CCOF-CuTPP-catalyzed one-pot asymmetric Henry reaction. (**e**) Effect of temperature on asymmetric reactions. Adapted from ref. [98] with permission, copyright 2019, Nature Publishing Group.

The work of Dong’s group has been crucial in advancing chiral COFs and asymmetric photocatalytic organic synthesis. Copper porphyrin is a common organic photothermal conversion material that can enhance the non-radiative transition of copper (II) [96,97]. Dong’s group reported several Cu(II) porphyrinyl-based chiral COFs for photo-assisted thermocatalysis [98,99,100,101]. In 2019, the group reported two metal nanoparticle-loaded chiral COFs (denoted Au@CCOF-CuTPP and Pd@CCOF-CuTPP) comprising a porphyrin-derived chiral COF [98]. The chiral COF was prepared by assembling copper tetrabromophenolphthalein (Cu-TBrPP) and the chiral organic monomer (*S*)-(+)-2-methylpiperazine (*S*-MP) in anhydrous 1,4-dioxane using Pd(PPh_4_)_3_ as a catalyst (Figure 9a). The crystalline COF was then subjected to successive solution impregnation and metal reduction steps to obtain the final metal nanoparticle-loaded chiral composite. Interestingly, both nanoparticle-loaded composites show effective photothermal conversion with a maximum temperature rise of 25–31 °C within 1080 s (Figure 9b,c). Meanwhile, Pd@CCOF-CuTPP can catalyze the asymmetric A^3^ coupling reaction by photothermal conversion, providing the desired products with yields of 68–98% and ee values of 90–98%. Under visible light, Au@CCOF-CuTPP can catalyze the asymmetric Henry reaction of benzyl alcohols and nitromethane with excellent yields (93–99%) and enantioselectivity (94–98% ee) (Figure 9d,e).

**Figure 10:**
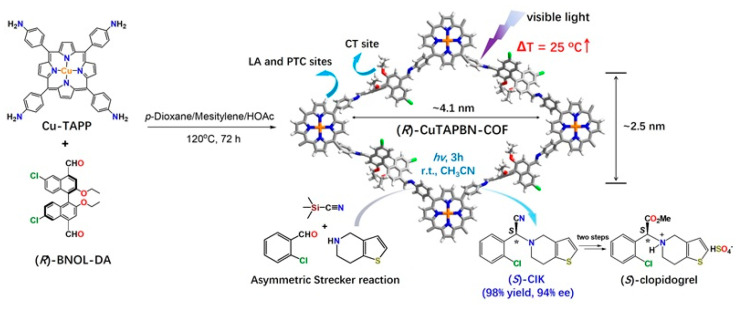
Synthesis of multifunctional chiral (*R*)-CuTAPBN-COF and its application in catalytic synthesis of drug intermediates. Adapted from ref. [101] with permission, copyright 2020, American Chemical Society.

Later, Dong’s group designed and synthesized the multifunctional chiral (*R*)-CuTAPBN-COF through the condensation reaction between 5,10,15,20-tetrakis-(4-aminophenyl)-porphyrin-Cu(II) (Cu-TAPP) and 6,6′-dichloro-2,2′-diethoxy-1,1′-binaphthyl-4,4′-dialdehyde ((*R*)-BINOL-DA) (Figure 10) [101]. It is noteworthy that the chiral control and catalytic centers of the (*R*)-CuTAPBN-COF are not on the same molecular body, a structural motif not dissimilar to the structure of enzymes. As an ADP receptor blocker, (*S*)-clopidogrel ((*S*)-CIK) is currently one of the best-selling antiplatelet and antithrombotic drugs in the world. Under visible light irradiation, (*R*)-CuTAPBN-COF can catalyze the one-step synthesis of (*S*)-CIK by a photothermally triggered three-component one-pot asymmetric Strecker reaction (Figure 10). Interestingly, the reaction can be carried out effectively under sunlight irradiation (the reaction system temperature is ~47 °C), providing (*S*)-CIK in up to a 70% yield and 92% ee within 3 h. Furthermore, the catalytic reaction has demonstrated excellent substrate scope. This work provides an experimental basis for the development of green energy-saving “windowsill” reactions based on chiral COF photocatalysis.

In 2022, Dong and Chen et al. reported another Cu(II)-metalated chiral COF using a similar method. The obtained (*R*)-CuTAPBP-COF contains both Lewis acid (copper porphyrin) and Brønsted acid (phosphoric acid) catalytic sites [100]. Under visible light irradiation, (*R*)-CuTAPBP-COF can catalyze the intermolecular asymmetric α-benzylation of aldehydes through photothermal conversion induced by visible light. For example, 4-(bromoethyl)pyridine reacts with propanaldehyde in methanol to produce (*R*)-2-methyl-3-(pyridine-4-yl)propane with a yield of 98% and with 95% ee. Moreover, this kind of photothermally catalyzed asymmetric reaction can also be effectively carried out under natural light.

**Figure 11:**
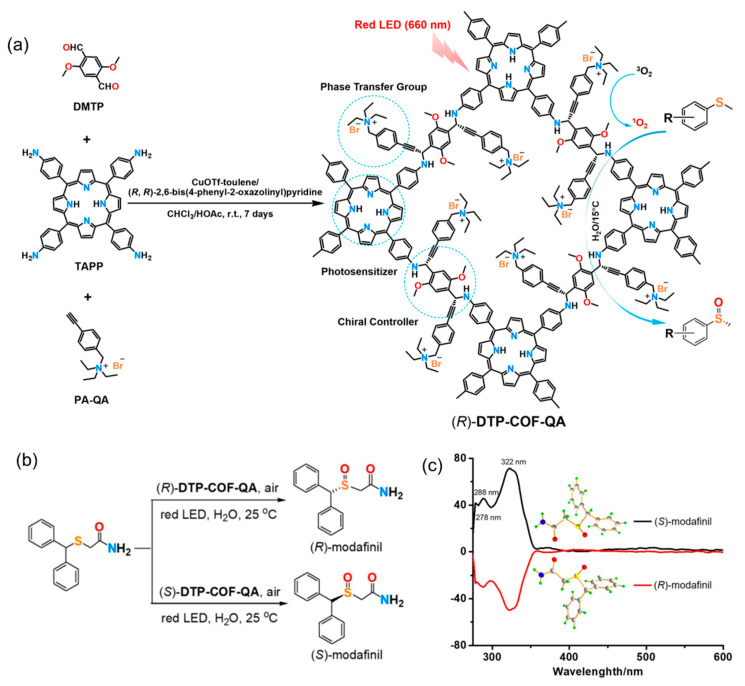
(**a**) Synthesis of quaternary ammonium bromide-decorated chiral photocatalyst (*R*)-DTP-COF-QA. (**b**) Photocatalytic synthesis of (*R*)- and (*S*)-modafinil by chiral DTP-COF-QA. (**c**) Circular dichroism spectra of (*R*)- and (*S*)-modafinil. Adapted from ref. [102] with permission, copyright 2022, American Chemical Society.

Although chiral COFs are increasingly used in photothermal asymmetric catalysis, their application in asymmetric photocatalysis remains scarcely documented. Recently, Dong’s group developed a quaternary ammonium bromide-decorated chiral photocatalytic system by integrating the functional photosensitizer 4-(10,15,20-triphenylporphyrin-5-yl)-aniline (TAPP), the phase transfer species quaternary ammonium bromide-decorated phenylacetylene (PA-QA), and chiral controller propargylamine into a chiral COF (denoted (*R*)-DTP-COF-QA) [102]. Under ambient conditions, (*R*)-DTP-COF-QA was synthesized from achiral 2,5-dimethoxyterephthaldehyde (DMTP), TAPP, and PA-QA through asymmetric A^3^-coupling polymerization in the presence of the chiral catalyst CuOTf-pybox (pybox = (*R*,*R*)-2,6-bis(4-phenyl-2-oxazolinyl)pyridine) (Figure 11a). (*R*)-DTP-COF-QA crystallizes in the chiral space group P4 and shows a broad absorption band covering the entire visible spectral range. Due to the synergistic effect between porphyrin, chiral propargylamine, and amphiphilic quaternary ammonium bromide, (*R*)-DTP-COF-QA can promote visible light-driven enantioselective photooxidation of sulfides to sulfoxides in water and in air with high activity and enantioselectivity (Figure 11b,c).

**Figure 12:**
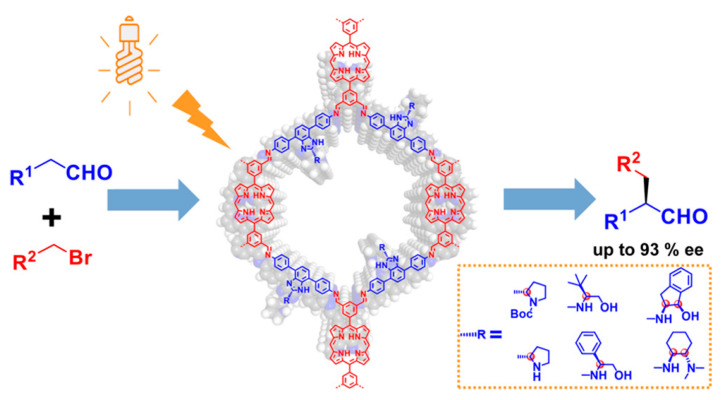
Chiral porphyrin-based COF-photocatalyzed asymmetric alkylation of aldehydes. Adapted from ref. [103] with permission, copyright 2023, American Chemical Society.

More recently, Zhao et al. reported a common bottom-up strategy for the successful synthesis of several photoactive chiral COFs [103]. They chose photoactive porphyrins as building blocks, immobilizing various secondary amine chiral catalytic centers on the COF pore walls by rationally designing benzimidazole linkers. In these chiral COFs, the porphyrin units act as antennas, capturing UV and visible light in the range of 250–1000 nm. At the same time, various secondary amine chiral functional groups were fixed on the channel wall of the chiral COFs through 1*H*-benzo[*d*]-imidazole building blocks, which weakened the influence of the flexible chiral functional groups on the crystallinity of the chiral COFs. Moreover, the reactants can easily reach the chiral catalytic site (the chiral secondary amine) through 1D nanotubes. Thus, well-designed chiral COFs can incorporate photoactive building blocks (porphyrin units) and chiral catalytic sites, resulting in high yields (97%) and excellent enantioselectivities (93%) in the photocatalytic asymmetric alkylation of aldehydes (Figure 12).

**Figure 13:**
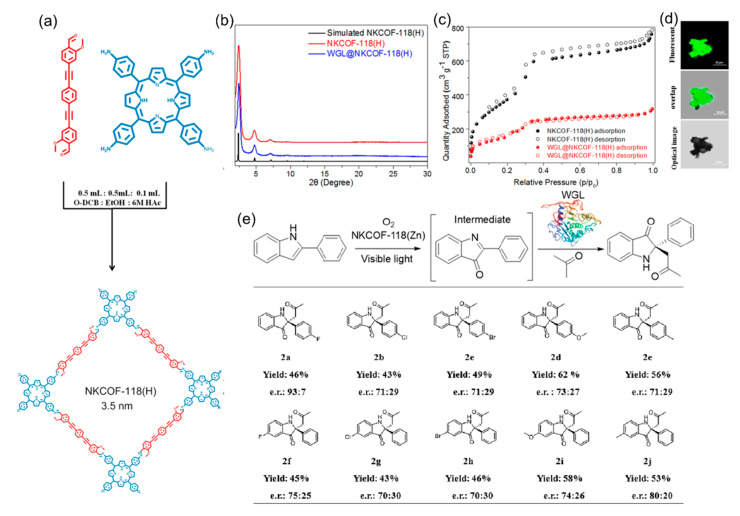
(**a**) Synthesis of mesoporous porphyrin-based COFs. (**b**) PXRD patterns of WGL@COFs. (**c**) N_2_ adsorption and desorption isotherms of NKCOF-118(H). (**d**) Confocal microscopy images of WGL@COFs. (**e**) Substrate scope of asymmetric Mannich reaction catalyzed by WGL@COFs. Adapted from ref. [104] with permission, copyright 2022, American Chemical Society.

Besides merging COFs with chiral catalytic sites like binaphthol derivatives and small organic molecules, integrating optically pure natural biomolecules such as enzymes and peptides into COFs with open channels also shows promise for the generation of photocatalysts of asymmetric organic transformations. For example, in 2022, Chen’s group designed and synthesized a photoenzymatic platform (WGL@COF) using mesoporous porphyrin-based COFs (NKCOF-118(M), M = H, Zn, Cu, and Ni) as solid carriers to immobilize wheat germ lipase (WGL) (Figure 13a) [104]. PXRD patterns, N_2_ adsorption and desorption isotherms, and confocal microscopy images demonstrate that WGL is successfully immobilized on the channel wall of porphyrin-based COFs (Figure 13b–d). Due to the proximity effect between the enzymes and porphyrin-based COF in one system, the resulting WGL@COFs exhibit good chemical stability and excellent photocatalytic activity in the asymmetric Mannich reaction under visible light irradiation (Figure 13e). Moreover, control experiments show that this asymmetric Mannich reaction cannot be achieved by porphyrin-based COFs or WGL independently. This work provides a simple and efficient method for producing highly efficient biomolecule-containing asymmetric photocatalysts by enzyme immobilization in photoactive COFs.

## 4. Conclusions

Homochiral COF materials possess diverse characteristics and applications and have been extensively studied in recent years. This review highlights the synthesis strategies, structural characteristics, and research progress related to chiral COFs in asymmetric photocatalysis. By applying three different synthesis strategies, many chiral COFs have been prepared. Post-modified synthesis can easily introduce chiral sites while preserving the framework of COFs. De novo synthesis is beneficial to obtain chiral COFs with permanent pores and defined chiral sites, but the protocol still presents significant challenges. Other methods besides solvothermal synthesis, like room temperature synthesis, microwave assisted reactions, and ionic thermal synthesis, should also be explored.

Due to their unique structural characteristics and ready tunability of their channels, chiral COFs show great potential in asymmetric photocatalysis. Typical asymmetric photocatalytic reactions like the Henry reaction, the Mannich reaction, and the alkylation of aldehydes have been demonstrated with excellent yields and enantioselectivities. However, compared with other porous materials, COFs have not yet been utilized in more complex and advanced asymmetric catalytic reactions. Their potential in asymmetric photocatalysis remains underdeveloped. Moreover, the fundamental mechanism of asymmetric photocatalysis using chiral COFs remains unclear. Although several COFs have recently been characterized by X-ray single-crystal diffraction, the structures of reported COFs are most usually determined by an analysis of powder X-ray diffraction and by taking into account the geometry principles developed in network chemistry. More detailed structural information is very useful for the study of the properties of COFs, so studying the crystallization process to prepare single crystals of chiral COFs and determining their atomic-level structure through a single-crystal X-ray diffraction analysis will provide more evidence for the elucidation of the catalytic mechanism of these materials. More 2D and 3D chiral COFs should be designed and synthesized to study their differences for asymmetric photocatalysis. When chiral COFs are used as photocatalysts, the dimensionality and pore size may particularly influence the asymmetric photocatalytic activity and catalyst sites of chiral COFs. In addition, new chiral COFs with achiral pore/channels need to be further investigated, and examples of their application to asymmetric photocatalysis are still unexplored.

## Data Availability

The research data are available by contacting the corresponding author.
